# Diet-Induced High Serum Levels of Trimethylamine-N-oxide Enhance the Cellular Inflammatory Response without Exacerbating Acute Intracerebral Hemorrhage Injury in Mice

**DOI:** 10.1155/2022/1599747

**Published:** 2022-02-16

**Authors:** Caizhen Li, Li Zhu, Yinming Dai, Zhiying Zhang, Leo Huang, Tom J. Wang, Peiji Fu, Yinuo Li, Jian Wang, Chao Jiang

**Affiliations:** ^1^Department of Neurology, The Fifth Affiliated Hospital of Zhengzhou University, Zhengzhou, Henan, China; ^2^Department of Psychology, University of Toronto, Toronto, Ontario, Canada; ^3^Winston Churchill High School, Potomac, Maryland, USA; ^4^Department of Anatomy, College of Basic Medical Sciences, Zhengzhou University, Zhengzhou, Henan, China

## Abstract

Trimethylamine-N-oxide (TMAO), an intestinal flora metabolite of choline, may aggravate atherosclerosis by inducing a chronic inflammatory response and thereby promoting the occurrence of cerebrovascular diseases. Knowledge about the influence of TMAO-related inflammatory response on the pathological process of acute stroke is limited. This study was designed to explore the effects of TMAO on neuroinflammation, brain injury severity, and long-term neurologic function in mice with acute intracerebral hemorrhage (ICH). We fed mice with either a regular chow diet or a chow diet supplemented with 1.2% choline pre- and post-ICH. In this study, we measured serum levels of TMAO with ultrahigh-performance liquid chromatography-tandem mass spectrometry at 24 h and 72 h post-ICH. The expression level of P38-mitogen-protein kinase (P38-MAPK), myeloid differentiation factor 88 (MyD88), high-mobility group box1 protein (HMGB1), and interleukin-1*β* (IL-1*β*) around hematoma was examined by western blotting at 24 h. Microglial and astrocyte activation and neutrophil infiltration were examined at 72 h. The lesion was examined on days 3 and 28. Neurologic deficits were examined for 28 days. A long-term choline diet significantly increased serum levels of TMAO compared with a regular diet at 24 h and 72 h after sham operation or ICH. Choline diet-induced high serum levels of TMAO did not enhance the expression of P38-MAPK, MyD88, HMGB1, or IL-1*β* at 24 h. However, it did increase the number of activated microglia and astrocytes around the hematoma at 72 h. Contrary to our expectations, it did not aggravate acute or long-term histologic damage or neurologic deficits after ICH. In summary, choline diet-induced high serum levels of TMAO increased the cellular inflammatory response probably by activating microglia and astrocytes. However, it did not aggravate brain injury or worsen long-term neurologic deficits. Although TMAO might be a potential risk factor for cerebrovascular diseases, this exploratory study did not support that TMAO is a promising target for ICH therapy.

## 1. Introduction

Intracerebral hemorrhage (ICH) is a devastating stroke with a high disability and mortality rate [1–4]. However, there is still a lack of effective treatment for patients with ICH [5–7]. Thus, it is essential to explore novel therapeutic targets for ICH. Furthermore, because inflammatory response plays a vital role in the progression of secondary brain injury after ICH [8], adjustment for immune-inflammatory response may represent a potential therapeutic target for ICH [9].

Studies have revealed that intestinal microbiota dysbiosis may influence the local and systemic immune response after stroke [10, 11]. The bidirectional communications between the central nervous system (CNS) and the gastrointestinal tract have been observed after stroke [10–13]. Evidence has indicated that stroke leads to dysbiosis of the intestinal flora [14]. In turn, intestinal flora disorder also induces intestinal immune status changes and might influence the systemic immunity and the prognosis of stroke [11, 13]. However, the mechanism for intestinal flora's regulation of the immune response is currently unknown. Thus, it is also necessary to explore the efficacy of intestinal flora metabolism in the pathophysiological process of acute stroke. Trimethylamine-N-oxide (TMAO) is previously considered a useless nitrogen-containing waste of intestinal flora metabolism [15, 16]. Recent studies showed that TMAO might be a new risk factor independent of traditional risk factors for ischemic stroke [15–20]. The positive association between plasma TMAO and the first or recurrent ischemic stroke has been suggested [21–23]. In addition, TMAO may aggravate endothelial cell dysfunction and promote the occurrence of atherosclerosis partly by activating P38-mitogen-protein kinase (P38 MAPK)/myeloid differentiation factor 88 (MyD88), activating and increasing the expression of high-mobility group box1 protein (HMGB1) [24–27]. Finally, studies revealed that high plasma TMAO levels on admission might be an independent predictor of stroke severity in patients with acute ischemic or hemorrhagic stroke [22, 28]. Consequently, the role of TMAO in the pathophysiological process of acute stroke warrants further research.

To explore whether TMAO can aggravate brain injury severity by promoting inflammatory response after acute stroke, this study observed the influence of high plasma TMAO levels on acute neuroinflammation, brain injury severity, and long-term neurologic function in mice with acute ICH. High serum TMAO concentrations were induced with a long-term choline diet. TMAO concentrations in the serum of mice were detected by ultrahigh-performance liquid chromatography-tandem mass spectrometry (UHPLC-MS/MS) at 24 h and 72 h after ICH. For early outcomes of ICH, we evaluated brain injury volume, brain swelling, brain edema, and inflammatory response in the acute phase of ICH. In addition, we evaluated myelin loss, brain atrophy, and residual lesion volume on day 28 for long-term effects. Neurologic deficits were assessed on days 1, 3, 7, 14, and 28 post-ICH.

## 2. Materials and Methods

### 2.1. Animals, ICH Model, and Treatment Regimen

The animal protocol was approved by the Animal Care and Use Committee of Zhengzhou University and the Fifth Affiliated Hospital of Zhengzhou University (K2019009). One hundred and twenty eight middle-aged male C57BL/6 mice weighing 25 to 35 g (10–12 months old) were purchased from Vital River Laboratory, Beijing, China. Animals were provided a regular chow diet (Xietong Pharmaceutical Bioengineering, Jiangsu, China) or a chow diet supplemented with 1.2% choline (Xietong Pharmaceutical Bioengineering, Jiangsu, China). Mice were kept under controlled temperature conditions (22 ± 2°C) under a 12-hour light/dark period with ad libitum access to water. These animal studies were reported by the ARRIVE guidelines [29].

The procedure for inducing ICH by collagenase injection in mice has been established in our laboratory [30, 31]. Briefly, mice were anesthetized by isoflurane (3.0% for induction, 1.0% for maintenance) and ventilated with oxygen-enriched air (20%: 80%) via a nose cone. Then, we injected the left caudate nucleus of mice with 0.075 U collagenase VIIs in 0.5 *μ*l saline to induce hemorrhage at the following stereotactic coordinates: 0.6 mm anterior and 2.0 mm lateral to the bregma and 3.2 mm in depth. Collagenase was delivered over 5 minutes, and the needle was left in place for an additional 10 minutes to prevent any reflux. The rectal temperature of the animals was maintained at 37.0 ± 0.5°C with a heating pad throughout the experimental and recovery periods. A postoperative neurological function score (NDS) below four was identified as a model failure; then, the mouse was excluded from the experiment.

Computer-generated random numbers were used to randomize mice into four groups: the sham+control group, sham+choline group, ICH+control group, and ICH+choline group [32, 33]. Mice in the control groups were given a regular chow diet (normal diet, Xietong Pharmaceutical Bioengineering, Jiangsu, China). Mice in the choline group were given a chow diet supplemented with 1.2% choline (choline diet, Xietong Pharmaceutical Bioengineering, Jiangsu, China) for six weeks before the operation and continuously given the same diet until the end of the experiment [20]. Investigators blinded to the treatment groups evaluated outcomes in all mice and performed data analysis.

### 2.2. Brain Lesion Volume, Swelling, Atrophy, and White Matter Damage

Similar to the clinical condition where a small penetrating artery ruptures, collagenase-induced ICH will bleed for a few hours [7, 30, 34, 35]. The hematoma volume will be stable relatively on day three after ICH [7, 30, 34]. Additionally, brain edema will be pronounced on day three after ICH in mice [30, 34, 36]. In this study, we detected hematoma volume and brain swelling on day three after ICH for early brain injury. To enhance the clinical relevance, we assessed neurologic function for 28 days and subsequently evaluated the long-term brain injury severity by detecting residual lesion volume, brain atrophy, and white matter damage according to the recommendations of The initial Stroke Therapy Academic Industry Roundtable (STAIR) and the requirements of the ARRIVE Guidelines for Reporting Animal Research in *vivo* [29, 37]. Mice were euthanized and underwent transcardial perfusion with 0.9% normal saline followed by 4% paraformaldehyde in 0.1 M phosphate buffer solution (PBS) after neurologic evaluation on day 3 or 28 post-ICH (for lesion volume on day 3, *n* = 8 per group; for residual lesion volume on day 28, *n* = 12 per group). The entire brain of each mouse was collected immediately, postfixed with 4% paraformaldehyde in PBS at 4°C overnight, dehydrated with 20% and 30% sucrose consecutively until they sank, embedded with the optimal cutting temperature compound, and then cut with a cryostat into 50 *μ*m sections at ten rostral-caudal levels that were spaced 360 *μ*m apart. The sections were stained with Luxol fast blue (for myelin) and Cresyl Violet (for neurons). SigmaScan Pro software (version 5.0.0 for Windows; Systat, San Jose, CA, USA) was used to quantify gray and white matter injuries. We calculated the lesion volume in cubic millimeters by multiplying the section thickness by the sum of the damaged areas of each section, as determined by the lack of specific staining [36, 38].

We quantified brain swelling (*n* = 8 mice/group) by calculating the percentage of hemispheric enlargement on the same sections on day three after ICH. Hemisphere enlargement (%) was expressed as [(ipsilateral hemisphere volume − contralateral hemisphere volume)/contralateral hemisphere volume] × 100% [39].

Brain atrophy (*n* = 12 mice/group) was quantified on day 28 after ICH according to the formula: [contralateral hemisphere volume − ipsilateral hemisphere volume)/contralateral hemisphere volume] × 100% [39, 40].

White matter damage (*n* = 12 mice/group) was quantified on day 28 after ICH. Light microscopy was used at the same exposure level to analyze three different sections from each mouse. Images from the four comparable fields around the residual lesion in each section were taken. The areas covered by the Luxol fast blue (LFB) stain were quantified with NIH ImageJ software, averaged, and then divided by the area of white matter to determine the degree of white matter damage [39, 41].

### 2.3. Brain Water Content

Mice (*n* = 6 per group) were euthanized on day three after ICH as previously described to determine brain water content [42]. Briefly, the brain was removed and divided into contralateral and ipsilateral hemispheres and cerebellum. The samples were weighed immediately to obtain the wet weight and then placed in an electric blast drying oven at 100°C for at least 24 hours to get the dry weight. The percentage of brain water content was calculated as [(wet weight–dry weight)/wet weight] × 100%.

### 2.4. Immunofluorescence

Mice (*n* = 6 mice/group) were euthanized and underwent transcardial perfusion with normal saline followed by 4% paraformaldehyde in 0.1 M PBS after neurologic evaluation on day three post-ICH. The entire brain of each mouse was collected immediately, postfixed with 4% paraformaldehyde in PBS at 4°C overnight, and dehydrated with 20% and 30% sucrose consecutively until they sank. Coronal brain sections of 30 *μ*m thickness were obtained with a freezing microtome (Leica, Germany) and were kept at −20°C. Sections were blocked in 5% bovine serum albumin in 0.01 M PBS for 60 minutes at room temperature and then were incubated with rabbit anti-ionized calcium-binding adapter molecule 1 (Iba-1, microglial marker; 1 : 1000; #019-19741, Wako Chemicals), rabbit anti-myeloperoxidase (MPO, neutrophil marker; 1 : 150; ab208670, Abcam), or rabbit anti-glial fibrillary acidic protein (GFAP, astrocyte marker; 1 : 200; 16825-1-AP, Proteintech) at 4°C overnight. After being washed three times with 0.3% Triton X-100 in 0.01 M PBS, the sections were incubated with AlexaFluor 488-conjugated goat anti-rabbit IgG (1 : 1000; 35552, Invitrogen) or AlexaFluor 594-conjugated goat anti-rabbit IgG (2 drops of/ml; B40925, Invitrogen) for 60 minutes at room temperature in the dark. After rinsing with dH_2_O, the sections were mounted with fluorescent mounting media and coverslipped [43, 44]. Negative controls consisted of brain sections processed the same way as the tests apart from the omission of the primary antibody incubation step. A fluorescence microscope (Nikon, Japan) was used to observe Iba1-, GFAP-, and MPO-immunoreactive positive cells under a 20x objective. ImageJ software (ImageJ 1.4, NIH, USA) was used to quantify Iba1, GFAP, and MPO immunoreactivity [30, 45]. In the perihematomal brain region, the numbers of Iba1- and MPO-immunoreactive cells at 12 locations per mouse were quantified. They averaged as positive cells per microscopic field (3 sections per mouse and four comparable fields per section) in selected sections with similar lesion areas [30]. We defined microglia and macrophages as activated if the cells were spherical, amoeboid, or rod-like in appearance, had a diameter of >7.5 *μ*m in at least one direction, with short and thick processes, and exhibited intense Iba1 immunoreactivity. Resting microglia/macrophages were characterized by small-cell bodies (<7.5 *μ*m in diameter) with long processes and weak Iba1 immunoreactivity [30]. GFAP immunoreactivity was quantified with fluorescence intensity, as previously illustrated [46, 47].

### 2.5. Western Blotting

Six brains from each treatment group were used to measure protein expression at 24 h post-ICH. Western blotting was carried out as described previously [48–50]. Brain tissue around the hematoma was homogenized in radioimmunoprecipitation assay (RIPA) lysis buffer (50 mM Tris (pH 7.4), 150 mM NaCl, 1% Triton X-100, 1% sodium deoxycholate, 0.1% SDS, and general protease and phosphatase inhibitors) and centrifuged at 14,000 g for 30 min at 4°C. The protein concentration in the supernatant was determined using bicinchoninic acid reagents (Beijing Solarbio Science & Technology Co., Ltd.). 45 mg proteins from each sample were separated by 10% sodium dodecyl sulfate-polyacrylamide gel electrophoresis (SDS-PAGE) and transferred onto polyvinylidene fluoride membranes with trans-blot apparatus (Bio-Rad, Hercules, CA). The membrane was blocked using 5% nonfat milk and incubated at 4°C overnight with the following primary antibodies: rabbit anti-P38-MAPK (1 : 1000, 9212, Cell Signaling Technology), rabbit anti-MyD88 (1 : 1000, 4283, Cell Signaling Technology), rabbit anti-interleukin- (IL-) 1*β* (1 : 1000, 12703, Cell Signaling Technology) and rabbit anti-HMGB1 (1 : 1000, ab18256, Abcam), rabbit anti-GAPDH (1 : 5000, 10494-1-AP, Proteintech), and *β*-actin (1 : 3000, 20536-1-AP, Proteintech). GAPDH and *β*-actin protein were used as a loading control. After three washes with 1x TBST, the blots were incubated with horseradish peroxidase-conjugated affinipure goat anti-rabbit antibody (1 : 5000, AS00001-2, Proteintech) for one hour at room temperature. The bands were probed with an ECL hypersensitive chemiluminescence reagent Kit (Thermo, 34095) and visualized with the Bio-Rad imaging system. Band densities of the protein immunoblot images were analyzed by ImageJ software (ImageJ 1.4, NIH, USA). Target protein levels were calculated as the ratio of the gray value of the target protein to the gray value of the corresponding GAPDH or *β*-actin [51].

### 2.6. Neurologic Function Evaluations

The neurologic deficits were assessed using the NDS and the corner turn test on days 1, 3, 7, 14, and 28 after ICH or sham surgery (*n* = 12/group) [52–55]. The NDS included the following six parts: body symmetry, gait, climbing, circling behavior, forelimb symmetry, and compulsory circling. Neurologic deficits were scored on a scale of 0 to 4 for each test, with 0 being no neurologic deficit and 4 being the most severe. For the corner turn test, the mouse was placed in an area with a 30° corner and allowed to turn to leave the area. Turns were recorded only if the mice were fully rotated along either wall. The test was repeated ten times for each mouse, and the percentage of left turns was recorded.

### 2.7. Quantitation of TMAO

The concentrations of TMAO in the serum of mice were measured at 24 h and 72 h post-ICH or sham surgery. Under deep isoflurane anesthesia of mice, blood samples were collected via retroocular puncture. The samples were centrifuged at 1500 rpm for 15 minutes at 4°C after standing for 2 h at 4°C; the supernatants were collected and stored at -80°C until analysis. After the vortex mixing of 50 *μ*l sample and 10 *μ*l 100 *μ*g/l D9-TMAO, the mixture was added into 440 *μ*l methanol to precipitate the protein and centrifuged at 13000 rpm for 30 minutes at 4°C. Then, 20 *μ*l supernatant was collected and mixed with 180 *μ*l 15% methanol in 0.5 ml EP to test the concentrations of TMAO.

For brain tissues, 40 mg brain homogenate in 0.1 M sterilized potassium phosphate buffer was mixed with 150 *μ*l of 0.1% formic acid in acetonitrile and 10 *μ*l of an internal standard. The mix was then centrifuged at 13000 rpm for 30 minutes at 4°C and then transferred the supernatant into a vial with a disposable glass insert. Finally, 20 *μ*l supernatant was collected and mixed with 180 *μ*l 15% methanol in 0.5 ml EP to test the concentrations of TMAO [56, 57].

UHPLC-MS/MS measured TMAO concentrations in serum and brain tissues in positive MRM mode, and multiple reaction monitoring was used to detect the following transitions: m/z 76 → 58 for TMAO and m/z 85 → 66 for d9-TMAO. The injection volume was 10 *μ*l, the mobile phase consisted of 0.1% formic acid in water (A phase) and 0.1% formic acid in acetonitrile (B phase) with the following gradient: 15% B phase at 3 min, then a linear gradient to 85% B phase from 3 min to 5 min, then maintained to 85% B phase from 5 min to 7 min, then linear gradient back to 15% B phase from 7 min to 9 min. Peak area under the chromatography curve was acquired and analyzed using Multiquant software (AB SCIEX).

### 2.8. Statistical Analysis

Sample sizes were determined with a power analysis based on one of our previous studies [30]. A power of 0.9 and a significance level of 0.05 were used to calculate the sample sizes. Based on mortality in one of our previous studies [30], the power analysis revealed that eight mice per group could acquire a significant difference in corrected lesion volume on day three. With our previous results reported as means and standard deviation of the means of the neurologic deficit score on day 28 [30], the power analysis showed that ten completed mice in each group would be sufficient to detect a significant difference in neurologic deficit on day 28. Sample size use for other variables is consistent with the recommendations of STAIR and the requirements of the ARRIVE Guidelines for Reporting Animal Research in *vivo* [29, 37]. SPSS 23.0 and GraphPad Prism 7.0 were used for the statistical analysis of this study. The quantitative data were expressed as mean ± standard deviation. The Kolmogorov–Smirnov test determined whether sample data were normally distributed. Student's *t*-test or *U* test was used to test the difference between the two groups. One-way analysis of variance test followed by the Bonferroni correction or nonparametric (Kruskal-Wallis test) were used for the examination of difference among multiple groups. Chi-square was used for the comparison of mortality of two groups. Repeated analysis of variance followed by the Bonferroni correction was used to detect changes in rectal body temperature, body weight, and NDS between treatment groups over time. *p* < 0.05 was considered to be statistically significant.

## 3. Results

### 3.1. Effects of Choline Diet on Serum Concentrations of TMAO in Mice

**Figure 1:**
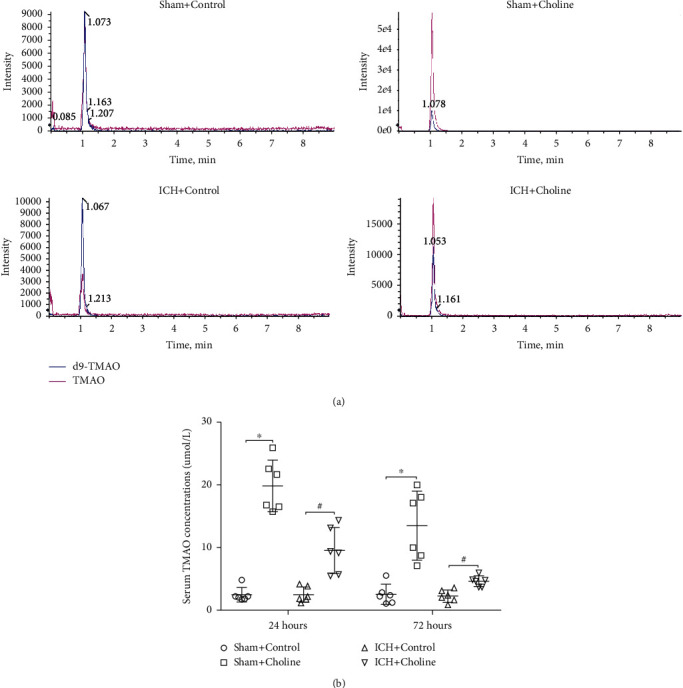
Choline diet remarkably increased the serum levels of TMAO in mice. (a) Representative results for the concentrations of TMAO in the serum of mice in each group detected by UPLC-MS/MS at 24 h and 72 h after sham operation or ICH. (b) Dot plots show the quantitative analysis of the concentrations of TMAO in the serum of mice that received a regular diet/choline diet at 24 h and 72 h after sham operation or ICH. ^∗^*p* < 0.05 compared with the sham+control group (*n* = 6 mice/group); ^#^*p* < 0.05 compared with the ICH+control group (*n* = 6 mice/group).

The results of UHPLC-MS/MS showed that choline diet significantly increased the serum concentrations of TMAO in mice received a choline diet when compared with those in mice that received a regular diet at 24 h and 72 h after sham operation (*n* = 6 mice/group, all *p* < 0.001, Figure 1). However, the serum concentrations of TMAO in mice with ICH tended to be lower than those of mice who underwent a sham operation at 24 h and 72 h after ICH or sham surgery (*n* = 6 mice/group, Figure 1). The serum concentrations of TMAO in mice with a choline diet were significantly higher than those with a regular diet at 24 h after ICH (*n* = 6 mice/group, *t* = 4.47, *p* = 0.001; Figure 1). Although a descending trend with time since ICH onset was also observed after ICH, the serum concentrations of TMAO were still higher in mice with a choline diet than mice with a regular diet at 72 h after ICH (*n* = 6 mice/group, *t* = 4.47, *p* = 0.001; Figure 1). We also tried to detect the content of TMAO in the brain tissues on day one after ICH or sham surgery. However, the content values of TMAO detected in the brain tissues were smaller than the minimum value of the standard curve (Supplementary Figure 1). Therefore, we did not compare their difference in brain tissues after ICH or sham surgery.

### 3.2. Effects of Choline Diet on Mortality, Weight, and Rectal Temperature

No mice died after sham surgery. In the collagenase-induced ICH model, the mortality of mice who received a choline diet (13.8%, 5 of 36) was not different from that of mice who received a regular diet (8.3%, 3 of 36, *χ*^2^ = 0.563, *p* = 0.453). The choline diet did not affect the rectal temperature at any time point during the research period (*n* = 12 mice/group, *F* = 0.052, *p* = 0.821; Supplementary Figure 2A). The decrease in the bodyweight of ICH mice lasts for 14 days. Percent change in body weight from the baseline did not differ between mice with a choline diet/regular diet on days 1, 3, 7, 14, and 28 (*n* = 12 mice/group, *F* = 0.057, *p* = 0.815; Supplementary Figure 2B).

### 3.3. Effects of Choline Diet-Induced High Serum Levels of TMAO on Brain Lesion Volume, Brain Swelling, and Water Content in Mice with ICH

**Figure 2:**
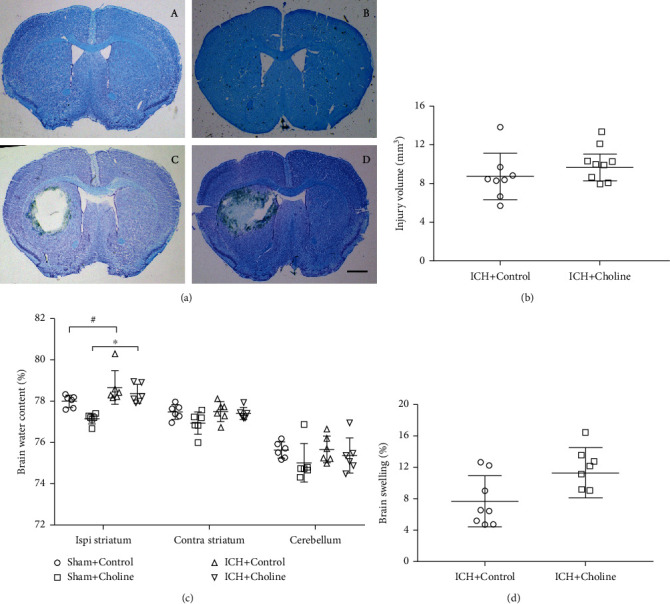
High serum levels of TMAO did not affect short-term brain injury after acute ICH. (a) Representative images of LFB/Cresyl Violet-stained brain sections on day 3 after sham operation or ICH, scale bar = 1 mm. A lack of staining indicates the lesion area; (A–D) The sham+control group, sham+choline group, ICH+control group, and ICH+choline group, respectively. (b) Brain lesion volume was measured with LFB/Cresyl Violet-stained brain sections. Quantification analysis revealed that brain lesion volume in mice fed with a choline diet was similar to that in mice fed with a regular diet on day three after ICH (*n* = 8 mice/group). (c) The water content in the ipsilateral striatum increased significantly regardless of regular diet or choline diet after ICH and TMAO did not affect the brain water content on day three after ICH; ^#^*p* < 0.05 compared with the sham+control group; ^∗^*p* < 0.05 compared with the sham+choline group (*n* = 6 mice/group). (d) The brain swelling in mice fed with a choline diet is similar to that in mice fed with a regular diet after ICH (*n* = 8 mice/group).

Brain lesson volume is closely related to the prognosis of ICH. Therefore, we observed whether the choline diet affected brain lesson volume on day three after ICH. We identified brain lesions lacking color on sections stained with LFB/Cresyl Violet. There was no significant difference in brain lesion volume between mice with a choline diet/regular diet on day three after ICH (*n* = 8 mice/group, *t* = 0.537, *p* = 0.514; Figure 2).

Brain swelling can cause brain injury and mortality in patients with ICH. Therefore, we calculated the degree of brain swelling from hemispheric enlargement on day three after ICH. We found no significant difference in brain swelling between mice with a choline diet/regular diet on day three after ICH (*n* = 8 mice/group, *U* = 16.0, *p* = 0.09; Figure 2).

We also evaluated brain water content in the ipsilateral and contralateral striatum and cerebellum with the dry-wet weight method on day three after ICH. We found that brain water content in the ipsilateral striatum, but not the contralateral striatum and cerebellum, of mice with ICH was significantly higher than that of mice underwent sham surgery regardless of regular diet or the choline diet (Kruskal-Wallis test value for the ipsilateral striatum was 17.414, *p* = 0.01; Kruskal-Wallis test values for the contralateral striatum and cerebellum were 6.810 and 2.741, respectively, *p* values for the contralateral striatum and cerebellum were 0.078 and 0.433; Figure 2). In addition, we also found that there was no significant difference in water content of the ipsilateral and contralateral striatum and cerebellum between mice with a choline diet/regular diet on day three (all *p* > 0.05, Figure 2).

### 3.4. The Effects of Choline Diet-Induced High Serum Levels of TMAO on the Expression of Inflammatory Factors around Hematoma in Mice with ICH

**Figure 3:**
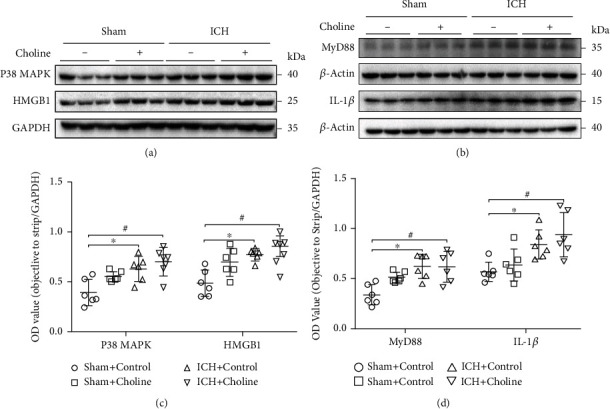
High serum levels of TMAO did not affect molecular inflammatory response in the hemorrhagic brain. (a, b) Representative western blot showing relative protein expression of P38 MAPK, MyD88, HMGB1, and IL-1*β* in brain lysates from the sham and ICH mice fed with a regular or choline diet 24 h after ICH. *β*-Actin and GAPDH were used as a loading control (*n* = 6 mice/group). (c, d) Dot plots show the quantitative analysis of P38 MAPK, MyD88, HMGB1, and IL-1*β* expressions in each group at 24 h after acute ICH (*n* = 6 mice/group; ^∗^*p* < 0.05 versus the sham+control group, ^#^*p* < 0.05 versus the sham+control group).

We detected the effects of choline diet on the relative expression of P38 MAPK, MyD88, HMGB1, and IL-1*β* around the hematoma with western blot analysis at 24 h after ICH or sham surgery. We found that there were significant differences in the expression of P38 MAPK (*F* = 7.429, *p* = 0.002), MyD88 (*F* = 6.973, *p* = 0.002), HMGB1 (*F* = 11.753, *p* < 0.001), and IL-1*β* (*F* = 6.821, *p* = 0.002) around hematoma among the four groups (*n* = 6 mice/group, Figure 3 and Supplementary Figure 3). ICH elevated the expression of P38 MAPK, MyD88, HMGB1, and IL-1*β* around the hematoma when compared with sham surgery at 24 h after ICH or sham surgery (all *p* < 0.05, Figure 3 and Supplementary Figure 3). Although the expression of P38 MAPK, HMGB1, and IL-1*β* around the hematoma increased slightly in mice that received a choline diet when compared with those of mice that received a regular diet at 24 h after ICH, the difference was not statistically significant (all *p* > 0.05, Figure 3 and Supplementary Figure 3). Choline diet also did not affect the expression of MyD88 when compared with mice that received a regular diet at 24 h after ICH (*p* = 1.0, Figure 3 and Supplementary Figure 3).

### 3.5. Effects of Choline Diet-Induced High Serum Levels of TMAO on the Activation of Microglia and Astrocytes and the Infiltration of Neutrophils in Mice with ICH

**Figure 4:**
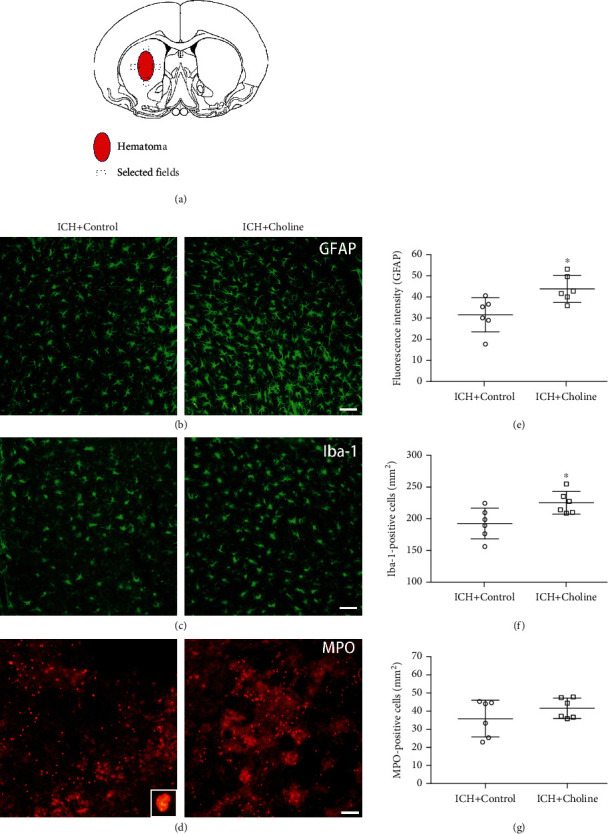
High serum levels of TMAO partly promoted the cellular inflammatory response in the hemorrhagic brain. (a) Schema chart of the selected fields for quantification of glial fibrillary acidic protein (GFAP), ionized calcium-binding protein-1 (Iba-1), and myeloperoxidase (MPO) in 3 comparable sections from each mouse. (b–d) Immunostaining for GFAP (b), Iba-1 (c), and MPO (d) in the perihematomal region on day three after acute ICH; scale bar = 50 *μ*m. Inset represents a higher magnification of MPO-positive neutrophils. (e–g) Dot plots show quantification analysis of activated astrocytes and microglia and infiltrated neutrophils (*n* = 6 mice/group, ^∗^*p* < 0.05 versus the regular diet group). Values are means ± SD.

Immunofluorescence staining of Iba-1 and GFAP was used to detect the effects of choline diet on the activation of microglia and astrocytes on day three after ICH. The activated microglia, defined as a combination of morphologic criteria and a cell body diameter cutoff of 7.5 *μ*m, were observed in the perihematomal region on day three [30]. The number of activated microglia around hematoma was significantly higher in mice that received a choline diet than in mice that received a regular diet on day three (*n* = 6 mice/group, *t* = 2.66, *p* = 0.024; Figure 4). We also observed the activation of astrocytes in the perihematomal area on day three. We found that the immunoreactivity of GFAP was more intense, and the processes of astrocytes were longer and thicker in the peri-ICH area than that in the contralateral side. Fluorescence intensity was used to quantify the activation of GFAP [58, 59]. The results revealed that the fluorescence intensity of GFAP was higher in mice that received a choline diet than that in mice that received a regular diet on day three (*n* = 6 mice/group, *t* = 2.93, *p* = 0.015; Figure 4). Immunofluorescence labeling of MPO was used to assess the infiltration of neutrophils after ICH. MPO-immunoreactive neutrophils were detected in the hemorrhagic striatum on day three after ICH. Choline diet increased serum TMAO levels but did not affect neutrophil infiltration compared with the regular diet on day three after ICH (*n* = 6 mice/group, *t* = 1.19, *p* = 0.26; Figure 4).

### 3.6. The Effects of Choline Diet-Induced High Serum Levels of TMAO on Long-Term Brain Injury in Mice ICH

**Figure 5:**
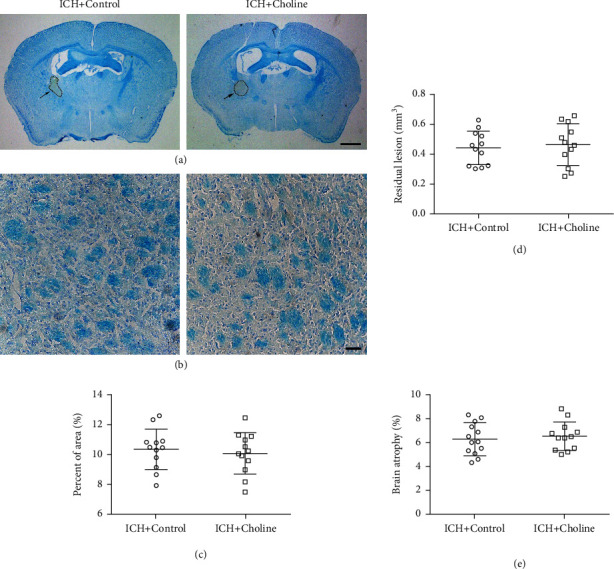
High serum levels of TMAO did not affect long-term brain injury after ICH. (a, b) Representative images of LFB/Cresyl Violet- or LFB-stained brain sections on day 28 after ICH; (a) scale bar = 1 mm; (b) scale bar = 50 *μ*m. (c) Quantitative analysis of white matter injury (*n* = 12 mice/group). (d, e) Dot plots show the quantitative analysis of lesion volume (d) and brain atrophy (e) in each group (*n* = 12 mice/group). Quantitative data are shown as mean ± SD.

We used LFB staining to label normal myelin on day 28 after ICH. We found that the white matter damage (loss of LFB-stained myelin) did not change significantly in mice that received a choline diet than in mice that received a regular diet on day 28 (*n* = 12 mice/group, *t* = 0.50, *p* = 0.63; Figure 5). Additionally, choline diet did not influence lesion volume (*n* = 12 mice/group, *t* = 0.44, *p* = 0.66; Figure 5) and brain atrophy (*n* = 12 mice/group, *t* = 0.51, *p* = 0.62; Figure 5) compared to regular diet on day 28, which indicates that choline diet increased serum TMAO levels but did not affect long-term brain injury.

### 3.7. The Effects of Choline Diet-Induced High Serum Levels of TMAO on Long-Term Neurologic Function in Mice with ICH

In this study, the corner turn test and the 24-point NDS were used to evaluate whether choline diet-induced higher serum TMAO levels significantly influenced neurologic function in mice with ICH on days 1, 3, 7, 14, and 28.

**Figure 6:**
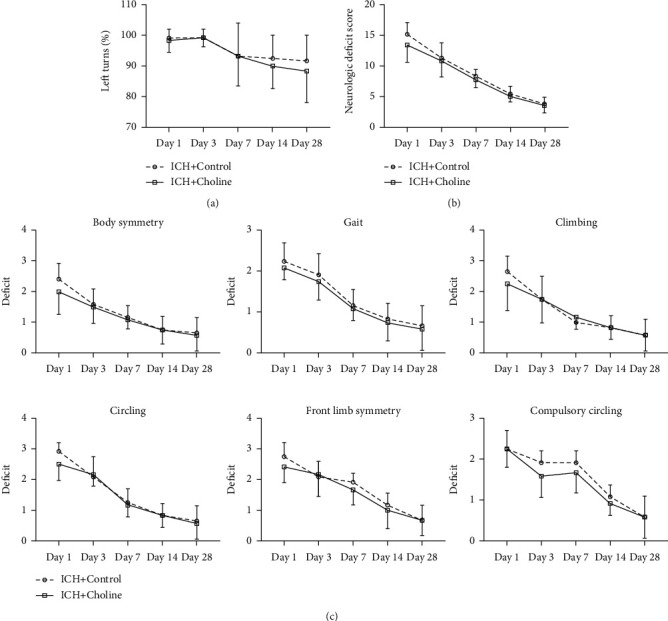
High serum levels of TMAO did not affect long-term neurologic deficits after ICH. Left turns (a), neurologic deficit scores (b), and neurologic deficit scores (NDS) for each of the individual tests (c) on days 1, 3, 7, 14, and 28 after ICH (*n* = 12 mice/group, all *p* > 0.05). Values are *means* ± *SD*.

The corner turn test revealed that mice turned left after ICH. However, the choline diet did not influence the corner turn test compared with the regular diet on days 1, 3, 7, 14, and 28 (*n* = 12 mice/group, *F* = 2.783, *p* = 0.109; Figure 6).

NDS assessment revealed that the neurologic deficits (body symmetry, gait, climbing experiment, voluntary rotation, forelimb symmetry, and forced rotation) were evident on day one and gradually recovered from days 3 to 28 after ICH. We also found that the choline diet did not influence the total NDS and individual NDS (body symmetry, gait, climbing, circling behavior, forelimb symmetry, and compulsory circling) at any test time point from days 1 to 28 after ICH (*F* values for total NDS, body symmetry, gait, climbing, circling behavior, forelimb symmetry, and compulsory circling were 3.458, 2.271, 1.878, 0.214, 1.347, 1.843, and 3.337, respectively; *p* values for total NDS, body symmetry, gait, climbing, circling behavior, forelimb symmetry, and compulsory circling were 0.076, 0.146, 0.184, 0.648, 0.258, 0.188, and 0.081, respectively; Figure 6).

## 4. Discussion

In this study, we found that the choline diet significantly increased the serum concentrations of TMAO compared with the regular diet at 24 h and 72 h after sham operation in mice. Possibly due to the influence of the ICH surgery on food intake and the short half-life of TMAO [25, 57], we found that the serum concentrations of TMAO tended to decrease in ICH mice receiving a choline diet when compared with that in sham-operated mice received a choline diet at 24 h and 72 h after ICH surgery or sham operation. However, the serum concentrations of TMAO were still higher in mice who received a choline diet than in mice who received a regular diet at 24 h and 72 h. We also found that the choline diet increased the activation of microglia and astrocytes around the hematoma. Still, it did not affect the expression of P38 MAPK, MyD88, HMGB1, and IL-1*β* around the hematoma in the acute phase of ICH. It also did not influence lesion volume, brain swelling, and brain water content in the acute stage of ICH. Moreover, we showed that the choline diet did not affect residual lesion volume, brain atrophy, brain white matter damage, and neurologic function on day 28. To our knowledge, this is the first study about the efficacy of long-term choline diet-induced high serum levels of TMAO on neuroinflammation, brain injury severity, and long-term neurologic function in a clinically relevant ICH model.

Knowledge gained about the origin and metabolic process of TMAO will be helpful for further exploration of TMAO's function. Studies have revealed choline, phosphatidylcholine, betaine, L-carnitine, and *γ*-butyryl betaine as precursors of TMAO [15, 16, 60, 61]. The synthesis of TMAO can be divided into the following two processes: firstly, nutrients, such as choline, phosphatidylcholine, betaine, and L-carnitine, are metabolized into trimethylamine by the intestinal flora; and then, after trimethylamine is transferred to the liver through the enterohepatic circulation, it is metabolized into TMAO by flavin monooxygenase (FMO), especially FMO3 [15, 16]. Previous research revealed that dietary supplementation with choline successfully increased the serum concentrations of TMAO in mice [15]. Consistently, our study also found that long-term dietary supplementation of mice with choline elevated the serum concentrations of TMAO at 24 h and 72 h after sham operation/ICH.

Age, gender, diet, intestinal flora, FMO3 activity, and renal excretion capacity are essential factors that influence the concentrations of TMAO in the serum [15, 16, 62–64]. Additionally, changes in the intestinal flora also have a profound impact on the serum levels of TMAO [65]. For example, research revealed that increased abundance of *Anaerococcus hydrogenalis*, *Clostridium asparagiforme*, *Clostridium hathewayi*, and *Edwardsiella tarda* significantly elevated the concentrations of TMAO in the host serum. On the contrary, an increased abundance of *Bacteroidetes*, *Prevotella*, and fecal bacteria reduced the concentrations of TMAO in the host serum [20]. Moreover, evidence revealed that opportunistic pathogenic bacteria, such as *Proteobacteria* and *Enterobacter*, increased. In contrast, the abundance of commensal or beneficial microbes, such as *Bacteroides* and *Prevotella*, decreased in patients with acute ischemic stroke or transient ischemic attack [65]. However, reports on changes in the intestinal flora of animals with acute ICH are rare. The impact of changes in intestinal flora on serum concentrations of TMAO warrants further research in animals with acute ICH.

The previous results about changes in the serum levels of TMAO are not consistent after acute ischemic stroke. One study found that the serum levels of TMAO were elevated in the acute phase of ischemic stroke [28]. However, most studies revealed that serum TMAO levels decreased with time since ischemic stroke onset in humans [65–68]. Our findings also showed that the serum levels of TMAO decreased in mice that received a choline diet after acute ICH than in mice that received a choline diet after sham surgery. Unfortunately, we did not document changes in food intake among the four groups after ICH or sham surgery in this study. Additionally, studies have revealed that TMAO injection-induced high serum levels of TMAO returned to the near baseline levels at about five hours in mice fed a regular chow diet, which suggests that the half-life of TMAO is very short *in vivo* [25, 57]. Therefore, it is necessary to study whether ICH surgery-associated reduction in food intake led to a decrease in serum concentrations of TMAO after acute ICH. Although the current understanding of TMAO's role in the CNS is limited, evidence indicates that TMAO, a small organic molecule, could penetrate the blood-brain barrier (BBB) [69]. Additional evidence also revealed that the concentrations of TMAO in the cerebrospinal fluid were higher in patients with neurodegenerative diseases such as Alzheimer's disease than in healthy controls [70, 71], which further supports the possibility that TMAO penetrates the BBB. To investigate whether TMAO can directly participate in the acute pathophysiological process of ICH, we measured TMAO's content in the brain homogenate extracts after acute ICH. Surprisingly, we found that the TMAO content in the brain tissues was undetectable (lower than the minimum value of the standard curve) in ICH mice regardless of a regular diet or a choline diet was given. The detection results of TMAO in brain tissues in this study may warrant further verification in further animal studies.

As a new risk factor independent of the traditional risk factors of ischemic cardiovascular and cerebrovascular diseases, TMAO can promote the occurrence and development of atherosclerosis by disturbing the reverse transport of cholesterol, and the synthesis of bile acids, and upregulating vascular endothelial cell inflammation [15, 16, 25, 72, 73]. The dysfunction of vascular endothelial cells is essential for the occurrence and development of atherosclerosis [74]. Studies have revealed that TMAO promoted vascular endothelial cell inflammation and advanced atherosclerosis by increasing the expression of the nuclear-factor kappa B (NF-*κ*B), nucleotide-binding oligomerization domain-like receptor family pyrin domain containing 3, IL-1*β*, and IL-18 [25, 72]. An additional study found that TMAO significantly reduced the expression of cell-cell junction proteins ZO-2, Occludin, and VE-cadherin through the HMGB1/TLR4 pathway, increasing endothelial cell permeability, and thus led to the dysfunction of endothelial cells [24]. Moreover, chronic or acute stimulation with TMAO resulted in the phosphorylation of P38 MAPK, an extracellular signal-related kinase, and the NF-*κ*B signaling cascade. It also increased the expression of inflammatory factors, such as IL-6, TNF-*α*, and intercellular cell adhesion molecule-1, and led to the dysfunction of vascular endothelial cells [25]. However, no study has researched the influence of TMAO on inflammatory response after acute stroke, including ischemic and hemorrhagic stroke.

The mass effect of hematoma causes the primary damage, whereas the inflammatory response induced by the disintegration products of hematoma contributes to the progression of secondary injury after ICH [30, 75]. Studies have revealed that the activation of microglia and astrocytes and the infiltration of neutrophils were essential for secondary brain injury after ICH [76–79]. HMGB1, defined as a cytokine rapidly released from the nucleus to the cytoplasm, promoted inflammatory response by activating microglia around the hematoma and aggravated brain injury after ICH in rats [80, 81]. The harmful role of P38 MAPK and its upstream signal MyD88 has also been confirmed in ICH's secondary brain injury process [82, 83]. As discussed previously, exposure to TMAO promoted chronic inflammatory response by increasing the expression of HMGB1, P38 MAPK, and IL-1*β* in endothelial cells [24–26, 72, 84]. To test the efficacy of TMAO on inflammatory response after acute ICH, we investigated choline diet-induced high serum levels of TMAO on the expression of HMGB1, MyD88, P38 MAPK, and IL-1*β* in the hemorrhagic brain of mice with acute ICH. Furthermore, we also explored the efficacy of choline diet-induced high serum levels of TMAO on the activation of microglia and astrocytes and the infiltration of neutrophils in the hemorrhagic brain in this study. Here, we showed that choline diet-induced high serum levels of TMAO significantly increased the activation of microglia and astrocytes but did not influence the infiltration of neutrophils around the hematoma in the acute stage of ICH. We also found that choline diet-induced high serum levels of TMAO did not affect the expression of HMGB1, MyD88, P38 MAPK, and IL-1*β* in the hemorrhagic brain after acute ICH in mice. This study illustrated that the choline diet-induced high serum levels of TMAO only partly promoted the inflammatory response in the hemorrhagic brain by activating microglia and astrocytes in the acute phase of ICH in mice.

As shown previously, TMAO promoted atherosclerosis partly by inducing an inflammatory response [24–26, 72]. TMAO also contributed to platelet hyperreactivity and enhanced thrombosis potential by augmenting Ca^2+^ release from intracellular stores [20]. Evidence has indicated that gut microbes increase platelet hyperreactivity and thrombosis potential through the generation of TMAO after acute ischemic stroke, which suggests that TMAO may participate in the pathophysiological process of acute ischemic stroke [20]. Some other studies showed that higher plasma TMAO levels on admission were an independent predictor of infarct volume and stroke severity in patients with acute ischemic stroke [22, 28, 85, 86]. Moreover, two clinical studies indicated that increased TMAO levels on admission predicted early neurological deterioration and unfavorable clinical outcomes on day 90 after acute ischemic stroke [86, 87]. However, contrary to the results of the previous studies on patients with ischemic stroke, we found that the difference in lesion volume and NDS was not significant between mice who received a choline diet/regular diet after acute ICH. It indicates that the choline diet increased the serum levels of TMAO but had no effect on brain injury severity or long-term neurologic function after ICH in mice. We speculate that the difference in the pathophysiological characteristics between ischemic and hemorrhagic stroke and the activity of platelets probably influenced by TMAO may explain why the results of this study are contrary to previous studies of ischemic stroke. Besides, TMAO has been viewed as a promoter of atherosclerosis; the severity of atherosclerosis should also be included as an essential factor when the efficacy of TMAO on stroke severity was evaluated in patients with ischemic stroke. As for hemorrhagic stroke, one clinical study showed that the increased TMAO level was independently correlated with 3-month function outcomes after ICH in patients [88]. Whether a choline diet for six weeks used in this study can mimic the dietary habit of patients with ICH before their symptom onset is currently unknown; the dynamic changes of serum TMAO levels and the predictive value of TMAO on the outcomes of patients with ICH warrant further study.

Additionally, the mass effect of hematoma may also have a profound influence on cerebral perfusion and cerebral blood flow by compressing perihematomal tissues or elevating intracranial pressure [89]. When the cerebral blood flow regulation is impaired, it causes a decrease in cerebral perfusion and cerebral blood flow [89]. However, no evidence has indicated whether choline diet-induced high serum levels of TMAO directly impact cerebral blood flow after acute stroke. As previously illustrated, TMAO can promote and aggravate atherosclerosis by disturbing cholesterol transport and promoting a chronic inflammatory response [15, 16, 25, 72, 73]. The severity of atherosclerosis may influence cerebral perfusion and cerebral blood flow in the perihematomal tissues or the whole brain after acute ICH. This exploratory study did not observe a positive relationship between choline diet or TMAO and brain injury or functional deficits after ICH. Further exploration should investigate the effects of choline diet-induced high serum levels of TMAO on cerebral perfusion and cerebral blood flow after ICH.

This exploratory study has limitations. One limitation of the current study is that we did not detect changes in the intestinal flora after acute ICH. Additional studies on the profile of intestinal flora further support the observed alterations in serum levels of TMAO. Moreover, broad-spectrum antibiotics and choline analogs such as 3,3-dimethyl-1-butanol reduced the serum levels of TMAO by regulating intestinal flora [90, 91]. Thus, the changes in serum concentrations of TMAO illustrated in this study can also be supported by research on the effects of broad-spectrum antibiotics or choline analogs on the profile of intestinal flora and the production of TMAO in ICH mice. As for the biological function of TMAO, previous studies have illustrated that TMAO-related chronic inflammatory response and abnormal lipid metabolism may promote the occurrence of atherosclerosis by inducing the dysfunction of vascular endothelial cells [24–26, 72]. However, we only observed that choline diet-induced high serum levels of TMAO partly aggravated the cellular inflammatory response in the hemorrhagic brain. We also observed that choline diet-induced high serum levels of TMAO did not influence brain injury severity or long-term neurologic function in mice with acute ICH. It seems that the 6-week choline diet-induced increase in the serum levels of TMAO is unlikely to exacerbate the pathology of acute ICH. One study showed that a 6-month choline diet predicted worse long-term outcomes [92]. Therefore, we suppose that the short-term diet regiment might be one of the reasons for the insignificant effect on ICH outcomes. It will be necessary to identify the lowest concentrations of TMAO in the serum or brain tissues that can aggravate brain injury with an intraperitoneal injection or intravenous tail injection of different concentrations of TMAO. FMO, and FMO3 in particular, is the primary metabolic enzyme of TMAO in the liver [93]. If the positive effects on ICH outcomes are present with high serum levels of TMAO, we plan to explore whether TMAO inhibition is protective in FMO3^−/−^ mice with acute ICH. Finally, it is worth investigating the impact of choline diet-induced high serum levels of TMAO on the late inflammatory response, brain repair, and long-term neurologic function recovery after ICH.

## 5. Conclusions

Although TMAO may promote atherosclerosis by inducing a chronic inflammatory response [24–26, 72], the impact of choline diet-induced high serum levels of TMAO on the acute ICH injury is inapparent. The causal relationship between the increased serum levels of TMAO induced by unhealthy eating habits and brain injury severity or prognosis of stroke warrants further research [28, 87, 88]. Moreover, large-scale clinical studies are warranted to verify whether there is a bias in recent studies about the positive association between TMAO and brain injury severity or prognosis of stroke in patients. Additional evidence on the causality between TMAO and stroke may help us further understand the pathophysiology of stroke in the future.

## Data Availability

All relevant data of this study are either included within the article and its supplementary files or are available upon request from the corresponding author.

## Abbreviations

TMAO: Trimethylamine-N-oxide
ICH: Intracerebral hemorrhage
P38 MAPK: P38-mitogen-protein kinase
MyD88: Myeloid differentiation factor 88
HMGB1: High-mobility group box1 protein
IL-1*β*: Interleukin-1*β*
LFB: Luxol fast blue
NDS: Neurological deficit score
CNS: Central nervous system
Iba-1: Calcium-binding adapter molecule 1
MPO: Myeloperoxidase
GFAP: Glial fibrillary acidic protein
UHPLC-MS/MS: Ultrahigh-performance liquid chromatography-tandem mass spectrometry
FMO: Flavin monooxygenase
BBB: Blood-brain barrier
NF-*κ*B: Nuclear-factor kappa B.

## Additional Points

*Highlight*s. (i) This study mimicked the impact of a high choline diet on patients with acute ICH. (ii) Choline diet remarkably increased TMAO serum levels in mice with acute ICH. (iii) Choline diet partly promoted neuroinflammation, probably via TMAO after ICH in mice. (iv) Choline diet did not aggravate brain injury and the outcome of ICH in mice. (v) This exploratory study did not support that TMAO can be targeted for ICH therapy.

## Supplementary Materials

Supplementary Materials Supplementary Figure 1: TMAO content detected in the brain tissues of mice with acute ICH. Supplementary Figure 2: effects of choline diet on body weight and rectal temperature of mice with ICH. Supplementary Figure 3: additional detection results for the expression of P38 MAPK, MyD88, HMGB1, and IL-1*β* on western blot. Supplementary File 1: raw western blot bands in Figure 3 and Supplementary Figure 3 and the raw immunofluorescence images in Figure 4.
