# Supplementary material for: Diet-Induced High Serum Levels of Trimethylamine-N-oxide Enhance the Cellular Inflammatory Response without Exacerbating Acute Intracerebral Hemorrhage Injury in Mice
Source: Oxid Med Cell Longev. 2022 Feb 16;2022:1599747. doi: 10.1155/2022/1599747 (PMC8886754; doi:10.1155/2022/1599747)
Supplement: Supplementary Materials — Supplementary Figure 1: TMAO content detected in the brain tissues of mice with acute ICH. Supplementary Figure 2: effects of choline diet on body weight and rectal temperature of mice with ICH. Supplementary Figure 3: additional detection results for the expression of P38 MAPK, MyD88, HMGB1, and IL-1β on western blot. Supplementary File 1: raw western blot bands in Figure 3 and Supplementary Figure 3 and the raw immunofluorescence images in Figure 4. [file 1599747.f2.docx]

**Diet-induced high serum levels of trimethylamine-N-oxide enhance the cellular inflammatory response without exacerbating acute intracerebral hemorrhage injury in mice**

**Supplementary figures**


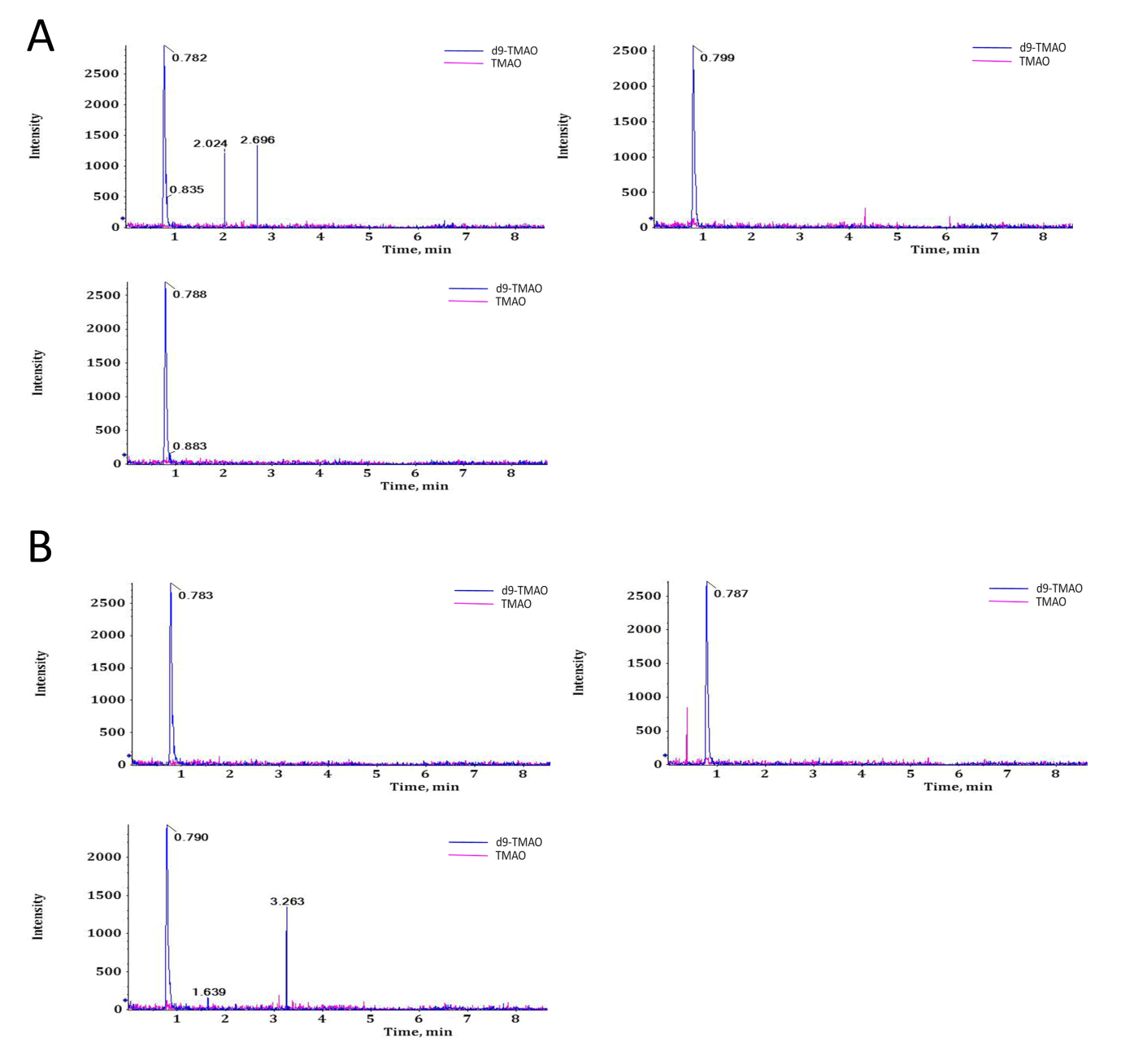


**Supplementary Figure 1. TMAO content was measured in the brain tissue of mice with acute ICH.** The TMAO content was measured in the brain tissue around the hematoma of mice on day one post-ICH (A: Results for three samples from mice who received a regular diet; B: Results for three mice who received a choline diet). The TMAO content detected in the brain tissues around the hematoma was lower than the minimum value of the standard curve.


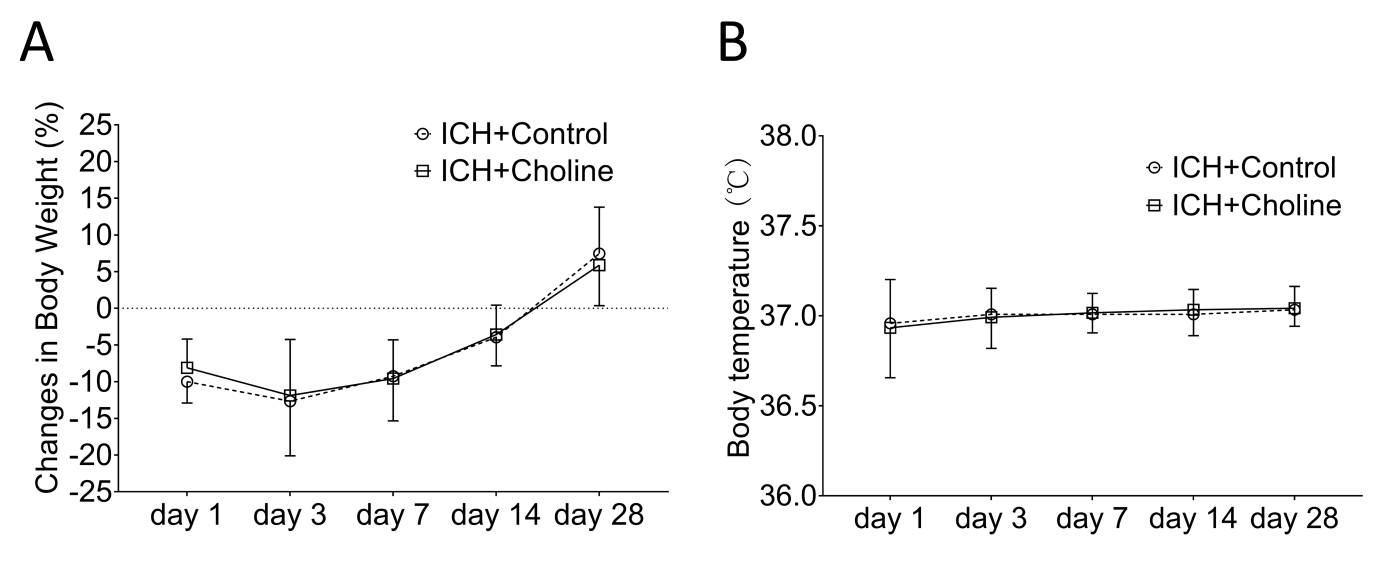


**Supplementary Figure 2. Effects of choline diet on body weight and rectal temperature of mice with ICH.** High serum levels of TMAO induced by the choline diet did not affect body weight (A) or rectal temperature (B) in mice on days 1, 3, 7, 14, and 28 after ICH. *n*=12 per group; Values are means ± SD.


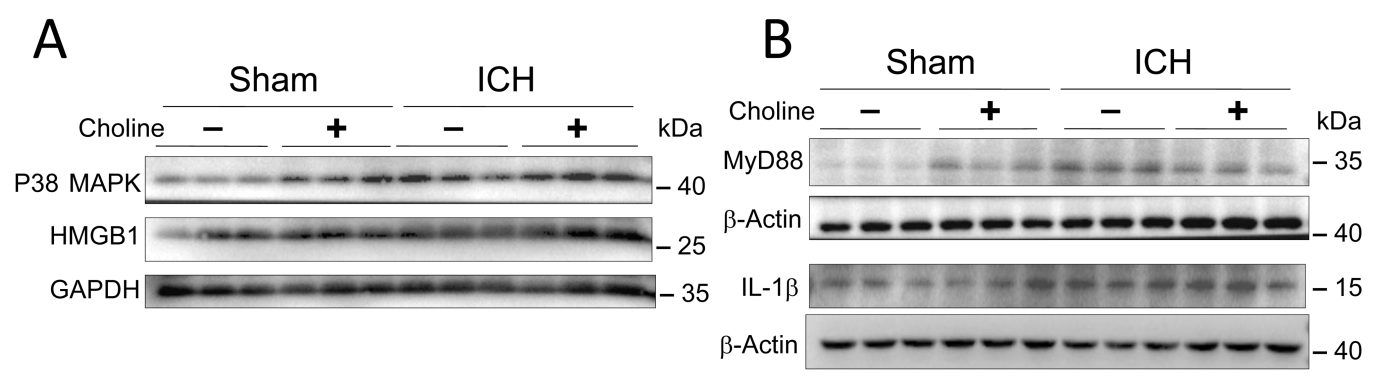


**Supplementary Figure 3. Additional results for the expression of P38 MAPK, MyD88, HMGB1, and IL-1β by Western blotting.** Results from 3 mice in each group (A. P38 MAPK and HMGB1; B. MyD88 and IL-1β). The bands in Figure 3 and this figure were analyzed to evaluate the effects of choline diet-induced high serum levels of TMAO on brain inflammatory response after acute ICH.

**Supplementary File 1**

**Raw Western blot bands in Figure 3**

P38 MAPK

**
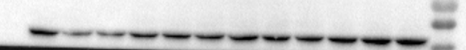
**

40 kDa

HMGB1

**
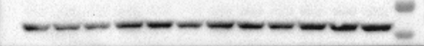
**

25 kDa

GAPDH

**
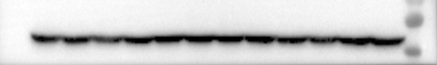
**

35 kDa

MyD88

**
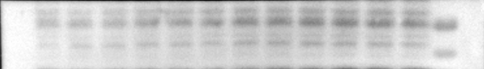
**

35 kDa

β-Actin

**
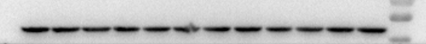
**

40 kDa

IL-1β

**
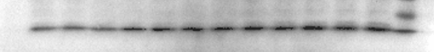
**

15 kDa

β-Actin

**
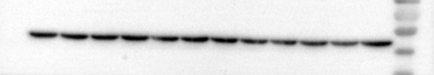
**

40 kDa

**Raw Western blot bands in supplementary figure 3**

P38 MAPK

**
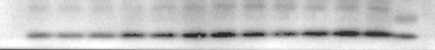
**

40 kDa

HMGB1

**
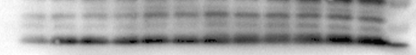
**

25 kDa

GAPDH

**
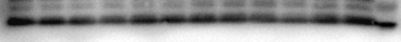
**

35 kDa

MyD88

**
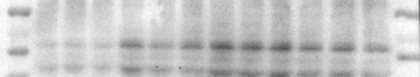
**

35 kDa

β-Actin

40 kDa

**
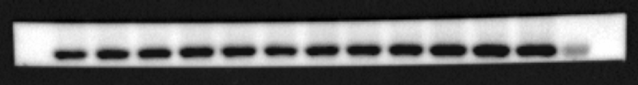
**

IL-1β

**
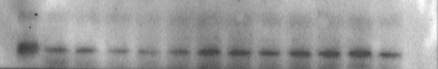
**

15 kDa

β-Actin

**
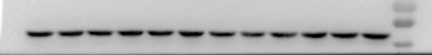
**

40 kDa

**The raw immunofluorescence images in Figure 4**

**GFAP (ICH+Control)**

**
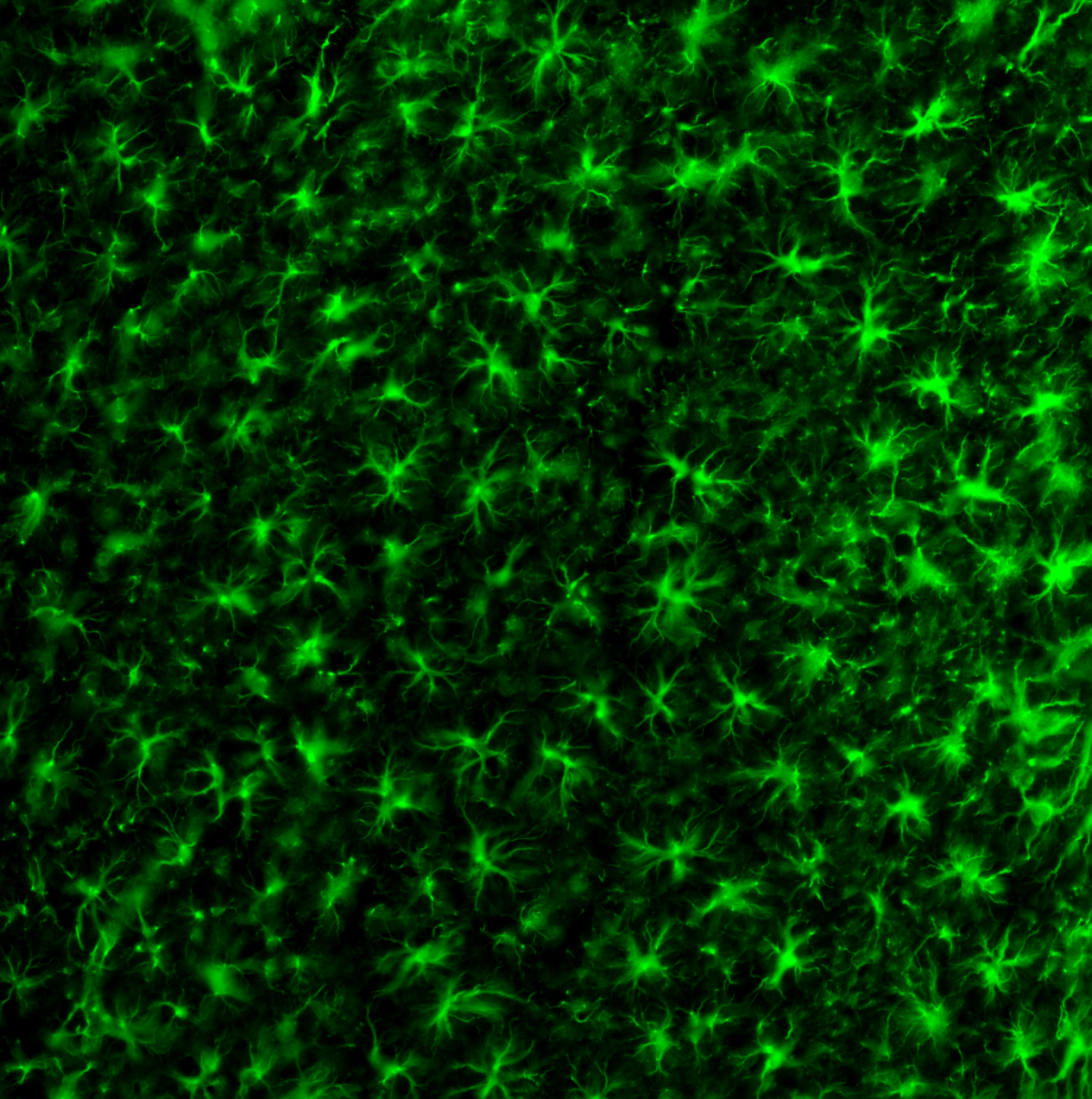
**

**GFAP (ICH + Choline)**

**
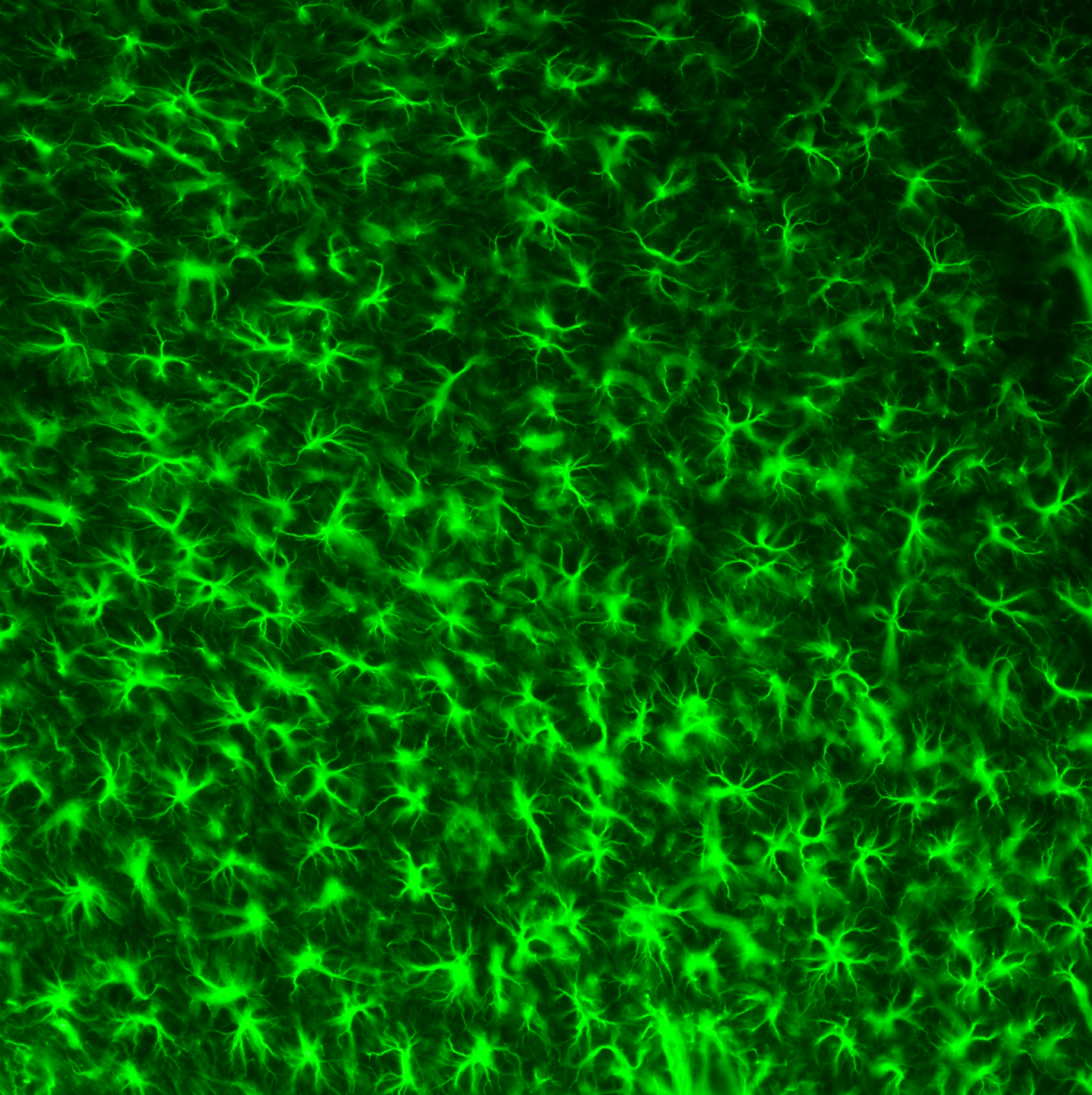
**

**Iba-1 (ICH + Control)**

**
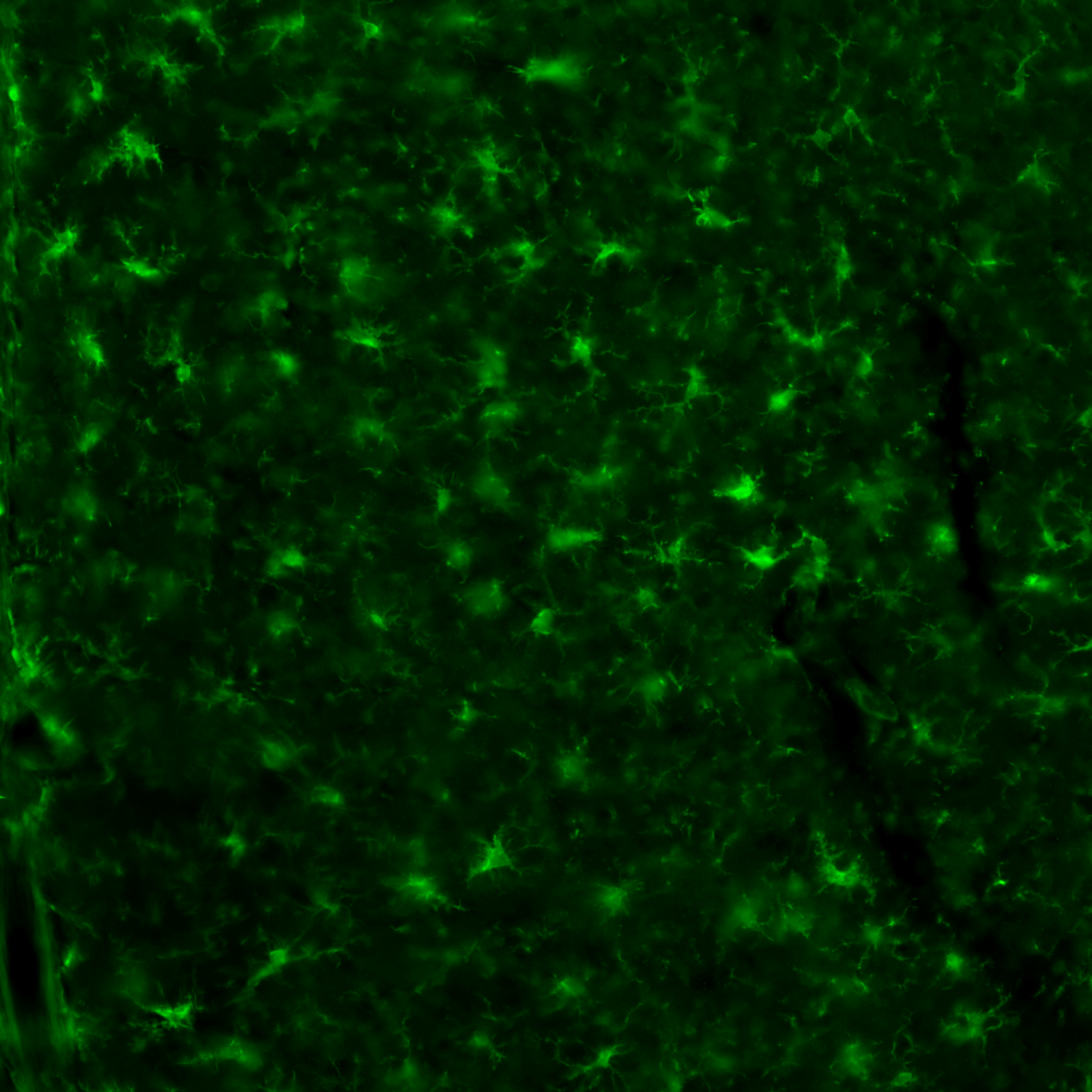
**

**Iba-1 (ICH + Choline)**

**
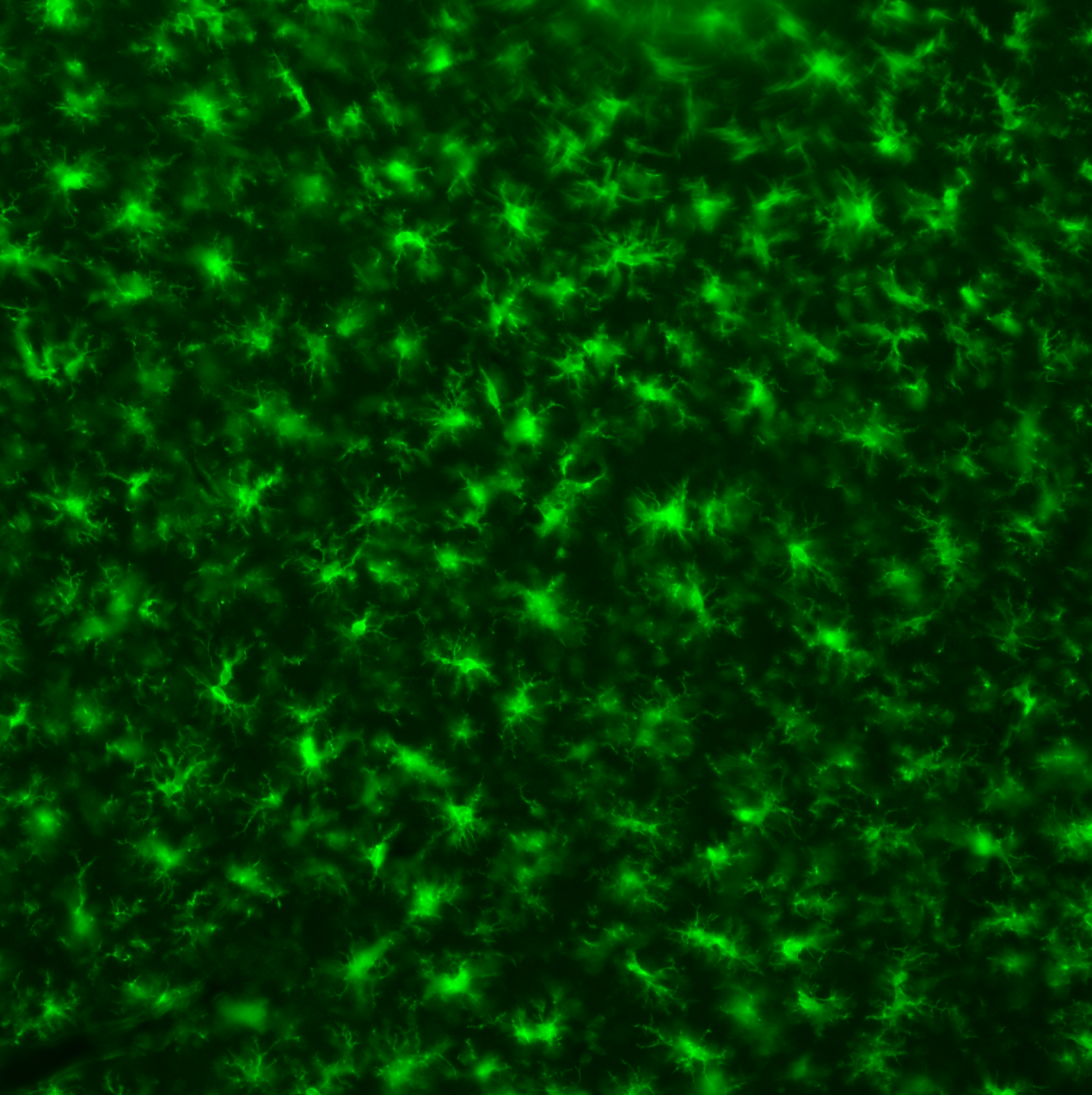
**

**MPO (ICH + Control)**

**
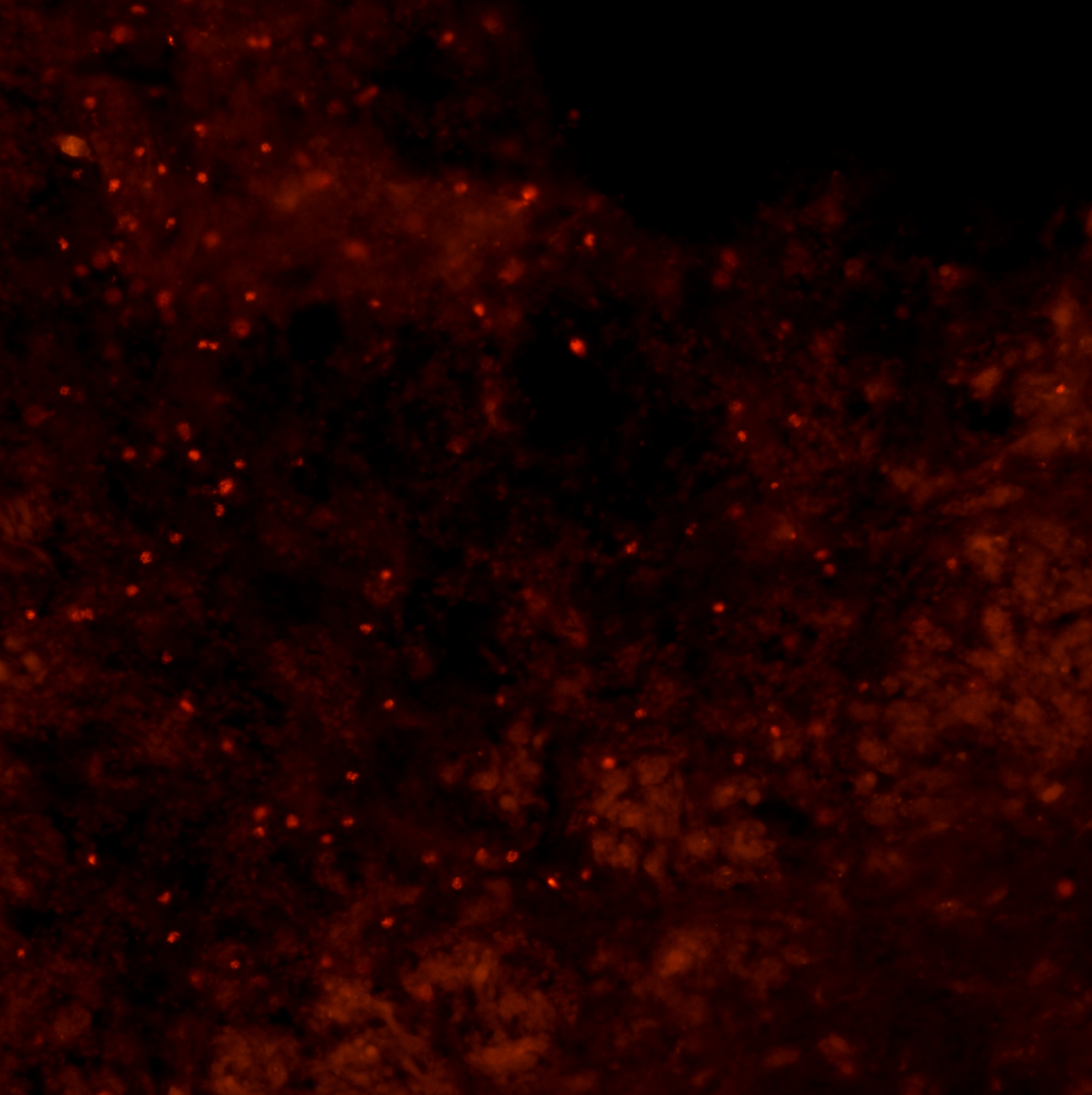
**

**MPO (ICH + Choline)**

**
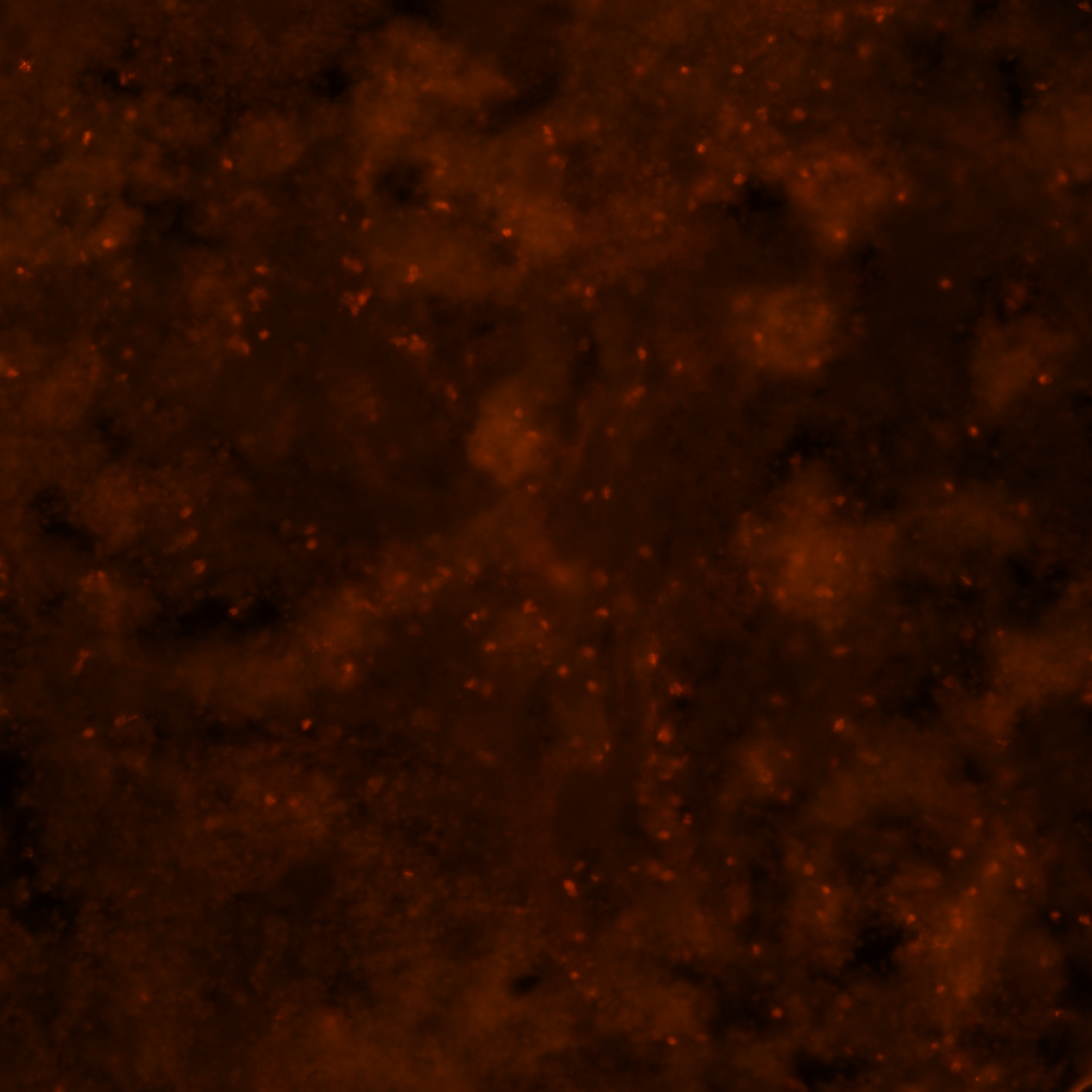
**
